# Glucagon in Pediatric Metabolic Disorders: Pathophysiology and Therapeutic Perspectives

**DOI:** 10.3390/pediatric17050104

**Published:** 2025-10-08

**Authors:** Giada Di Pietro, Francesco Chiarelli, Angelika Mohn

**Affiliations:** Department of Paediatrics, University G. d’Annunzio, 66100 Chieti, Italy; giada.dipietro@studenti.unich.it (G.D.P.); chiarelli@unich.it (F.C.)

**Keywords:** glucagon, obesity, diabetes

## Abstract

Over the past century of research, it has become increasingly evident that glucagon should no longer be regarded solely as a counter-regulatory hormone to insulin. Its role in the pathophysiology of metabolic disorders—including diabetes, obesity, and non-alcoholic fatty liver disease—appears to be critical. Hyperglucagonemia is a common feature across several metabolic conditions, not only in adults but also in pediatric populations, suggesting that glucagon may represent both a pathogenic factor and a potential therapeutic target in metabolic disease. Accordingly, therapeutic strategies have been developed that either inhibit or enhance glucagon activity, depending on the clinical context, and some of these approaches are being applied in pediatric care as well. This review aims to provide a comprehensive overview of the pathophysiological role of glucagon in metabolic diseases, synthesizing recent findings that support novel hypotheses for the management and prevention of these conditions.

## 1. Introduction

(i) Historical background

In 2023, the centenary of glucagon’s discovery was commemorated. Initially identified in 1923 as a contaminant of early insulin preparations, glucagon was only later recognized as a hormone, with its endocrine role being established in the 1950s. Since then, both animal and human studies have confirmed its essential role in glucose metabolism and have highlighted its equally important functions in amino acid and lipid metabolism [1].

Traditionally regarded as an anti-insulin hormone, glucagon was soon employed in clinical practice to treat insulin-induced hypoglycemic coma in individuals with type 1 diabetes mellitus (T1DM). Over the past decades, research has increasingly shifted toward understanding the broader physiological roles of glucagon, particularly its involvement—together with pancreatic α-cells—in the pathophysiology of type 2 diabetes mellitus (T2DM) and obesity [2].

These advances have redefined glucagon not merely as a counter-regulatory hormone but as a central player in the regulation of whole-body energy homeostasis. However, progress in this field was long constrained by technical limitations in the accurate measurement of circulating glucagon levels [3], a challenge that has recently been addressed through the development of high-specificity ELISA methods [4].

(ii) Aims of this review

Despite a century of investigation, glucagon remains incompletely understood. The growing body of evidence points to complex and context-dependent actions of this hormone, particularly in pediatric metabolic disorders. The aim of this review is to synthesize recent findings on the pathophysiological role of glucagon and to critically reinterpret existing knowledge in order to highlight novel hypotheses and therapeutic perspectives. In doing so, we hope to contribute to the identification of innovative research directions and improved strategies for the prevention and management of metabolic disease.

## 2. Necessary Physiology Hints

Glucagon has traditionally been recognized as an antagonist of insulin due to its opposing metabolic effects on glucose homeostasis. Specifically, glucagon directly regulates glucose metabolism through three principal mechanisms: in the liver, it enhances glucose production by stimulating glycogenolysis and gluconeogenesis [5]; in adipose tissue, it promotes lipolysis, leading to the release of free fatty acids and the subsequent formation of ketone bodies in the liver [6], both contributing to a net increase in circulating glucose levels. In contrast, glucagon also acts on pancreatic β-cells by inhibiting insulin secretion, thereby playing a key role in maintaining glucose balance. Glucagon exerts its effects by binding to the glucagon receptor (GCGR), which is primarily expressed in hepatocytes, adipocytes, and β-cells [7]. However, GCGR has a broad tissue distribution, which underlies the hormone’s diverse physiological actions. In addition to its classical targets, GCGR expression has been identified in the kidneys, heart, lymphoblasts, spleen, brain, adrenal glands, retina, and gastrointestinal tract [8].

Glucagon also modulates blood glucose levels indirectly through renal mechanisms by enhancing water reabsorption and glomerular filtration, which may in turn increase glucose reabsorption [9]. Nonetheless, the role of glucagon extends beyond glucose regulation. For instance, postprandial increases in glucagon concentrations suggest its involvement in meal-induced satiety [10]. Although the mechanisms underlying glucagon-induced satiety remain incompletely understood, it has been hypothesized that vagal afferent fibers in the hepatic branch relay signals to the central nervous system [5]. Furthermore, glucagon contributes to weight loss by slowing gastric emptying and increasing energy expenditure [11]. The physiological functions of glucagon in other GCGR-expressing tissues—such as the retina, heart, and gastrointestinal tract—remain largely unclear and warrant further investigation.

### 2.1. Glucagon and Liver–Acell Axis

**Figure 1:**
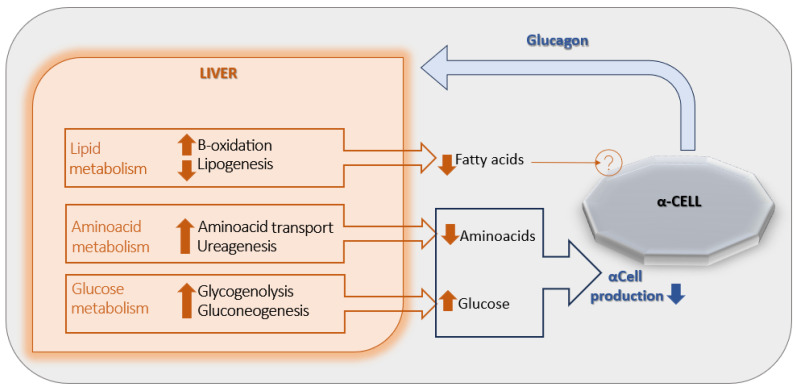
The liver-α-cell-axis in health. Adapted with permission of American Diabetes Association Ref. [13]; 3 Jun 2022; Richter, Michael M.; Galsgaard, Katrine D. [The Liver-α-Cell Axis in Health and in Disease, American Diabetes Association, 2022. Copyright and all rights reserved. Material from this publication has been used with the permission of American Diabetes Association.

The primary target organ of glucagon is the liver, where a regulatory feedback mechanism known as the “liver–alpha cell axis” has been described [Figure 1] [12]. Specifically, the glucagon-induced stimulation of glycogenolysis and gluconeogenesis results in a net increase in hepatic glucose output, which in turn exerts negative feedback on α-cell glucagon secretion. In addition, glucagon enhances hepatic uptake and turnover of amino acids, leading to a reduction in circulating amino acid levels and promoting ureagenesis—another mechanism contributing to the suppression of glucagon release. Glucagon also stimulates hepatic β-oxidation while inhibiting lipogenesis, thereby decreasing circulating free fatty acids (FFAs) concentrations. However, the potential role of reduced FFAs in the inhibition of glucagon secretion remains unclear, as no definitive mechanism has yet been identified [6].

### 2.2. Hyperglucagonemia: The Main Character

Metabolic disorders have traditionally been attributed to an absolute or relative deficiency of insulin, a perspective known as the insulin-centric theory [14]. However, in 1978, Unger and colleagues challenged this view by proposing the bihormonal regulation theory, based on emerging evidence regarding the role of glucagon [15]. According to their findings, certain metabolic abnormalities observed in diabetes—such as increased lipolysis, enhanced proteolysis, and impaired glucose utilization—are directly linked to insulin deficiency. In contrast, other disturbances, including reduced glycogen synthesis, enhanced ketogenesis, and increased hepatic glycogenolysis and gluconeogenesis, are attributed to glucagon excess.

Toward the end of the 20th century and the beginning of the 21st, the glucagonocentric theory—originally envisioned by Unger—gained support. Key evidence includes the observation that mice lacking the GCGR do not develop hyperglycemia despite insulin deficiency, and that hyperglucagonemia is consistently observed across all forms of diabetes in humans. These findings suggest that glucagon excess is necessary for the development of hyperglycemia [16].

Under physiological conditions, hypoglycemia is the primary stimulus for glucagon secretion. Paradoxically, individuals with diabetes exhibit elevated glucagon levels even in the presence of hyperglycemia. Until recently, this phenomenon was explained primarily by the loss of tonic inhibition exerted by insulin on α-cells, reflecting the traditional model of unidirectional paracrine signaling from β- to α-cells [17].

Until the early 2000s, α-cell influence on β-cell function was considered negligible, in part due to the predominance of studies using rodent islets, in which α-cells are less abundant than in human islets [18]. More recently, a more nuanced understanding has emerged, recognizing a complex intra-islet vascular architecture that allows for bidirectional signaling and integration with the exocrine pancreas. This has led to the recognition of an active and dynamic role for α-cells in both physiological and pathophysiological states, culminating in the concept of α- to β-cell cross-talk within the islet microenvironment [19].

### 2.3. The Role of the Inter-Cellular Cross-Talk

Both glucagon and insulin receptors are expressed on α- and β-cells, supporting the existence of a reciprocal regulatory relationship between these two cell types. Insulin exerts a tonic inhibitory effect on glucagon secretion by α-cells through direct interaction with the insulin receptor; consequently, a reduction in insulin levels leads to increased glucagon secretion [20]. Notably, GCGRs are more abundantly expressed on β-cells than insulin receptors are on α-cells, and it has been demonstrated that glucagon can directly stimulate insulin secretion [21].

Furthermore, under hyperglycemic conditions, β-cells in close proximity to α-cells secrete greater amounts of insulin compared to β-cells lacking such interactions, highlighting the importance of intra-islet cellular communication [22]. Studies have also shown that individuals with T2DM exhibit elevated α-cell-to-β-cell mass ratios, possibly reflecting a compensatory mechanism whereby α-cells support β-cell insulin secretion [23]. While glucagon exerts some of its effects via GCGRs, it appears to stimulate insulin secretion primarily through activation of the glucagon-like peptide 1 (GLP-1) receptor expressed on the β-cell surface [24].

## 3. Hyperglucagonemia: The Common Feature

Although type 1 and type 2 diabetes mellitus (T1DM and T2DM) have distinct pathogenetic mechanisms, both conditions share a common feature: hyperglucagonemia, whose pathogenic role has long been underestimated. A hallmark of both T1DM and T2DM is the absence of postprandial suppression of glucagon, resulting in persistent hypersecretion [25]. Notably, elevated glucagon responses have also been observed in individuals with subtle impairments in glucose metabolism who do not meet the diagnostic criteria for diabetes, such as during an oral glucose tolerance test (OGTT) [26].

Several mechanisms may underlie hyperglucagonemia, and although it can be challenging to delineate clearly between metabolic disorders—many of which exist along a continuum—identifying the predominant mechanism in each condition may help tailor therapeutic strategies and improve clinical outcomes, as summarized in Table 1.

### 3.1. Hyperglucagonemia in Obesity and NAFLD

Nonalcoholic fatty liver disease (NAFLD) is the most prevalent chronic liver disorder in children and adolescents and is recognized as an early risk factor for the development of obesity and T2DM [27]. Emerging evidence suggests that hyperglucagonemia is more strongly associated with obesity and NAFLD than with diabetes itself; elevated fasting glucagon levels have been observed even in individuals with obesity and normal glucose tolerance [28].

**Figure 2:**
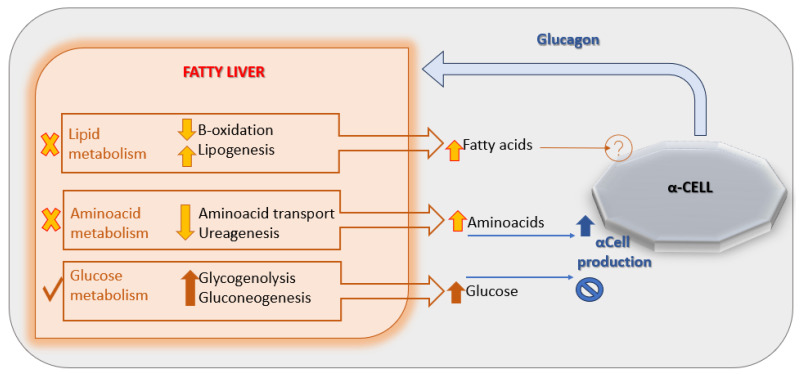
The liver-α-cell-axis in disease. Adapted with permission of American Diabetes Association Ref. [13]; 3 Jun 2022; Richter, Michael M.; Galsgaard, Katrine D. [The Liver-α-Cell Axis in Health and in Disease, American Diabetes Association, 2022]. Copyright and all rights reserved. Material from this publication has been used with the permission of American Diabetes Association.

A leading hypothesis proposes that NAFLD induces hepatic resistance to glucagon by disrupting the liver–alpha cell feedback axis [Figure 2], leading to increased circulating amino acid levels. These amino acids, in turn, stimulate α-cells to secrete glucagon, perpetuating hyperglucagonemia [29]. Supporting this model, a 2020 study demonstrated impaired glucagon-mediated amino acid turnover in the liver of individuals with obesity and NAFLD compared to lean, non-steatotic controls [30].

Given this causal link, plasma glucagon concentration may serve as a useful biomarker for identifying pediatric patients at higher risk of developing NAFLD [31].

### 3.2. Hyperglucagonemia in Obesity and T2DM

In metabolic disorders such as T2DM and obesity, impaired incretin production appears to play a key role in the pathogenesis of hyperglucagonemia. In this context, a study was conducted in individuals aged 10 to 18 years with obesity and varying degrees of glucose tolerance—ranging from impaired glucose tolerance (IGT) to overt T2DM—and compared to controls with normal glucose tolerance. The authors reported that, compared to controls, obese individuals with IGT exhibited reduced GLP-1 levels concomitant with elevated postprandial glucagon concentrations, as well as increased fasting glucagon levels in parallel with reduced fasting GLP-1 levels [32]. These alterations became more pronounced as glucose tolerance worsened, highlighting the significant contribution of incretin dysregulation to glucagon excess.

Given this evidence, GLP-1–based therapies may offer a more rational approach than metformin in treating T2DM, particularly in the context of concurrent hyperglucagonemia. Furthermore, chronic hyperglycemia has been shown to upregulate hepatic GCGR expression while simultaneously impairing downstream signaling, indicating the development of hepatic glucagon resistance [33]. Additionally, it has been hypothesized that the pathophysiology of T2DM may involve mutations in the gene encoding GCGR, further supporting a role for altered glucagon signaling in disease progression [34,35].

### 3.3. Hyperglucagonemia and T1DM

In light of the documented interaction between α- and β-cells, individuals with type 1 diabetes mellitus (T1DM) exhibit increased glucagon levels as a consequence of insulin deficiency and the resulting loss of tonic inhibition normally exerted by β-cells on α-cells. Glucagon plays a particularly critical role in the development of diabetic ketoacidosis (DKA), a life-threatening complication of T1DM [36]. In the context of insulin deficiency, glucagon activity predominates, promoting the transport of FFAs from the circulation into hepatic mitochondria, where they undergo β-oxidation to produce acetyl-CoA. This acetyl-CoA is subsequently used for ketone body synthesis [37].

Importantly, a distinction must be made between ketogenesis under physiological conditions, such as prolonged fasting—where it serves as an adaptive energy source—and the pathological ketogenesis observed in uncontrolled T1DM. In the latter case, ketone production results from metabolic dysregulation driven by insulin deficiency and glucagon excess and does not represent a regulated energy-producing mechanism [38,39].

DKA is associated with significant morbidity due to the accumulation of ketone bodies, which contribute to oxidative stress and inflammation. These effects predominantly impact cardiomyocytes, erythrocytes, and endothelial cells, thereby increasing the risk of both acute and long-term diabetic complications [40]. Moreover, elevated plasma ketone concentrations have been implicated in the downregulation of insulin receptor expression on the cell surface, thereby exacerbating insulin resistance [41].

Given that increased glucagon secretion during DKA contributes to metabolic deterioration alongside insulin deficiency, therapeutic strategies targeting hyperglucagonemia—rather than focusing solely on hyperglycemia and insulin replacement—may offer additional clinical benefit.

## 4. Therapeutic Perspectives in Metabolic Disorders

Hyperglucagonemia and α-cell hyperplasia are established contributors to metabolic dysfunction [42]. However, recent findings indicate that α-cells, via intra-islet paracrine signaling, may support β-cell function. The elevated glucagon levels seen in metabolic diseases could represent an adaptive mechanism to sustain energy balance and β-cell activity [43]. Whether hyperglucagonemia in the context of metabolic disease is pathogenic or compensatory remains unresolved [44].

Given glucagon’s central role in these disorders, therapeutic strategies increasingly focus on modulating its secretion or action, tailored to the specific condition and disease stage, especially in pediatric populations where early intervention may be crucial.

### 4.1. Glucagon Antagonism

In patients with hyperglycemia treated with insulin, glucagon excess contributes—at least in part—to poor glycemic control and may therefore be targeted through inhibition of glucagon secretion or action [45]. Antagonism of the GCGR has been proposed as a pharmacological strategy for the treatment of both T1DM and T2DM, and can be achieved through several approaches, including small-molecule receptor antagonists, monoclonal antibodies (mAbs) targeting GCGR, and antisense oligonucleotides designed to downregulate receptor expression [46].

Notably, GCGR-targeting mAbs have also been shown to promote β-cell regeneration by inducing the transdifferentiation of a subset of pancreatic α-cells or δ-cells into insulin-producing β-cells [47]. In clinical studies, a single dose of REMD-477 (Volagidemab), a GCGR monoclonal antibody, significantly reduced insulin requirements in patients with T1DM and improved glycemic control, without inducing serious adverse effects [48]. However, the available data remain limited, and further investigation is needed to validate efficacy and safety in broader patient populations.

### 4.2. The Multi-Effectiveness of GLP-1 Analogues

Glucagon-like peptide-1 analogues (GLP-1As) are now well-established and widely used agents for the treatment of obesity, and they have demonstrated superior efficacy compared to insulin and metformin in the management of T2DM. Furthermore, they may have potential as adjunctive therapy in T1DM.

The therapeutic strength of GLP-1As lies in their pleiotropic actions. They enhance glucose-dependent insulin secretion, inhibit glucagon secretion, promote pancreatic β-cell survival, growth and regeneration, slow gastric emptying, and reduce food intake. Notably, GLP-1As have also been approved for use in the pharmacological treatment of pediatric obesity [49].

It is reasonable to hypothesize that, even in the presence of GCGR mutations affecting β-cell signaling, the interaction between glucagon and the GLP-1 receptor (GLP-1R) remains functionally intact. As such, GLP-1As may offer therapeutic advantages that extend beyond the limitations of GCGR antagonism.

### 4.3. GLP-1A in T2DM

Currently, first-line therapies for T2DM in children over 10 years of age and adolescents include lifestyle modifications (diet and physical activity), as well as pharmacological treatment with insulin and metformin. GLP-1 receptor agonists (GLP-1As) are recommended as second-line agents.

The incidence of juvenile-onset diabetes (JOD) is rising in parallel with the increasing prevalence of obesity among adolescents [50]. Compared to adult-onset T2DM, JOD is associated with several distinct and more severe clinical features: greater impairment of pancreatic β-cell function—exacerbated by insulin resistance related to obesity and puberty—along with higher rates of both microvascular and macrovascular complications, despite a shorter disease duration. In addition, metformin, the standard first-line therapy, demonstrates a higher failure rate in this population [51].

Given these challenges, GLP-1As are likely to be increasingly employed earlier in the treatment course, potentially prior to the initiation of insulin, due to their dual benefits on weight reduction and glycemic control, as well as their inhibitory effects on glucagon secretion. Supporting this approach, a recent study demonstrated that once-weekly treatment with dulaglutide significantly improved glycemic control over 26 weeks compared to placebo in youth with T2DM already receiving metformin and/or insulin therapy [52].

### 4.4. GLP-1A in T1DM

In T1DM, residual β-cell function is typically minimal or entirely absent. As a result, GLP-1 receptor agonists (GLP-1As) are not expected to stimulate endogenous insulin secretion in these patients. Beyond glycemic control—the primary goal of insulin therapy—two important challenges in the management of T1DM are weight gain and the paradoxical increase in glucagon levels, which is often refractory to exogenous insulin administration [53].

A study demonstrated that the addition of a GLP-1A to insulin therapy resulted in improved glycemic control, reduced body weight, lower daily insulin requirements, and, notably, a significant reduction in both total and postprandial glucagon levels [54]. Furthermore, other authors have reported that postprandial glucagon concentrations tend to increase progressively with disease duration in T1DM, and this rise correlates positively with worsening glycemic control and further loss of β-cell function [55].

Interestingly, if GLP-1 levels were to increase in parallel with glucagon, despite GLP-1’s known inhibitory effect on glucagon secretion, this would suggest a discrepancy between the effects of physiological versus pharmacological GLP-1 levels—highlighting the therapeutic potential of GLP-1As in modulating glucagon activity. These findings provide a renewed rationale for the use of GLP-1As as adjunctive therapy to insulin in T1DM.

Supporting this hypothesis, a recent trial showed that liraglutide exerts an inhibitory effect on ketogenesis through the suppression of glucagon secretion [56]. Additionally, another study demonstrated that liraglutide not only significantly reduces postprandial glucagon excursions in a dose-dependent manner but also suppresses fasting plasma FFAs concentrations, thereby attenuating ketogenesis in patients with T1DM [57].

## 5. Conclusions and New Challenges

Hyperglucagonemia is a central pathogenic factor not only in all forms of diabetes but also in obesity. Its multifactorial etiology includes insulin deficiency, resistance to glucagon receptor (GCGR) signaling, dysregulation of incretin secretion, and disruption of the liver–alpha cell axis. Among these, hepatic steatosis—which is highly prevalent in obese pediatric patients—likely plays a pivotal role in promoting hepatic glucagon resistance. Importantly, hyperglucagonemia may serve as an **early biomarker** for the development of metabolic diseases in the pediatric population, offering a valuable tool for **targeted prevention strategies**.

A critical challenge for pharmacological research is balancing glucagon’s beneficial metabolic effects—such as regulation of body weight and lipid metabolism—with its potential to raise blood glucose levels. In this regard, novel dual and triple agonists that combine glucagon with incretin hormones have emerged as promising therapeutic options for both type 2 diabetes and obesity [58,59]. Notably, the GIP/GLP-1 receptor agonist tirzepatide has received FDA approval for T2DM treatment and has demonstrated superior efficacy over semaglutide in promoting weight loss among patients with obesity [60].

Looking forward, a **paradigm shift in therapeutic strategies** may be warranted. Current treatments focusing primarily on insulin and metformin might not fully address the complex pathophysiology of diabetes. Considering glucagon as a therapeutic target—even in conditions like diabetic ketoacidosis—could enhance treatment efficacy. Current therapies and future therapeutic proposals are listed in Table 1.

Reframing the therapeutic approach, especially in pediatric populations, has the potential to provide a more **comprehensive and effective management** of metabolic diseases.

## Figures and Tables

**Table 1:** Causes of hyperglucagonemia, current therapies and future therapeutic proposals.

Main Causes of Hyperglucagonemia	Metabolic Pathologies	Current Therapy	Therapeutic Innovation
(1) **Lack of suppression from insulin deficit**	T1DM	Insulin	GLP-1a GCGR-antagonists
(2) **Role of incretins**	T2DM—OBESITY	Life-style intervention Metformin GLP-1a	Combined GIP/GLP-1, GCGR-antagonists
(3) **Liver glucagon receptor resistance**	T2DM—OBESITY	Life-style intervention metformin GLP-1a	Combined GIP/GLP-1 GCGR-antagonists
(4) **Altered liver-alpha cell axis**	NAFLD	Life -style intervention	GLP-1a

## Data Availability

No new data were created or analyzed in this study. Data sharing is not applicable to this article.

## Abbreviations

The following abbreviations are used in this manuscript:

T1DM	Type 1 Diabetes Mellitus
T2DM	Type 2 Diabetes Mellitus
FFAs	Free Fatty Acids
NAFLD	Non-alcoholic Fatty Liver Disease
GCGR	Glucagon Receptor
GLP1	Glucagon-Like Peptide
DKA	Diabetic Keto-Acidosis
JOD	Juvenile-Onset Diabetes
DKA	Diabetic Keto Acidosis
